# Endovascular Repair of a Penetrating Axillary Artery Injury

**DOI:** 10.21470/1678-9741-2018-0226

**Published:** 2019

**Authors:** Abdulmajeed Altoijry, Thamer Nouh, Ahmed Alburakan, Magdi Ibrahim, Talal A Altuwaijri

**Affiliations:** 1 Division of Vascular Surgery, Department of Surgery, College of Medicine, King Saud University, Riyadh, Saudi Arabia.; 2 Trauma and Acute Care Surgery Unit, Department of Surgery, College of Medicine, King Saud University, Riyadh, Saudi Arabia.

**Keywords:** Case Reports, Gunshot Wounds, Axillary Artery - Injuries, Vascular System Injuries, Angioplasty

## Abstract

We report a 16-year-old boy who sustained a gunshot injury on his upper left side of the chest that resulted in an injury to the left axillary artery and was treated with endovascular repair. An endovascular repair has been increasingly accepted for the management of hemorrhage in critically ill trauma patients; using covered endovascular stents provides an alternative modality for both controlling hemorrhage and preserving flow.

## INTRODUCTION

Vascular injuries affecting the upper limbs more frequently occur due to penetrating than blunt trauma^[1,2]^. Among this class of injuries, axillary artery injuries are the most uncommon. Although rare, axillary artery injuries must be managed with prudent consideration to prevent complications such as hemorrhage, ischemia, and pseudo-aneurysm^[3]^. Surgical management is an extensive strategy and requires aggressive dissection with a high risk of injury to surrounding structures. Endovascular management has been found to reduce the operative time, cost, and exposure to general anesthesia, local dissection, and potential injury to other structures^[4]^. Here, we report a successful endovascular management of a vascular injury in the upper limb. Consent was obtained from the patient to publish the obtained images and his clinical history.

## CASE REPORT

A 16-year-old boy was admitted with a gunshot injury on the upper left side of the chest. He received initial treatment in another hospital while in shock and hypoxemic with a Glasgow coma scale score of 14. Thereafter, he was transferred to our facility with stable hemodynamics following resuscitation and placement of a chest tube. The gunshot entry point was approximately 1 cm below and lateral to the midclavicular line, and the exit point was just lateral to the scapular spine posteriorly, leaving a non-expanding and non-pulsatile chest hematoma. Bilateral radial, ulnar, and brachial pulses were palpable. The patient showed signs of brachial plexus injury and was not able to extend his wrist. Furthermore, he experienced impaired sensation in his upper left limb.

**Fig. 1:**
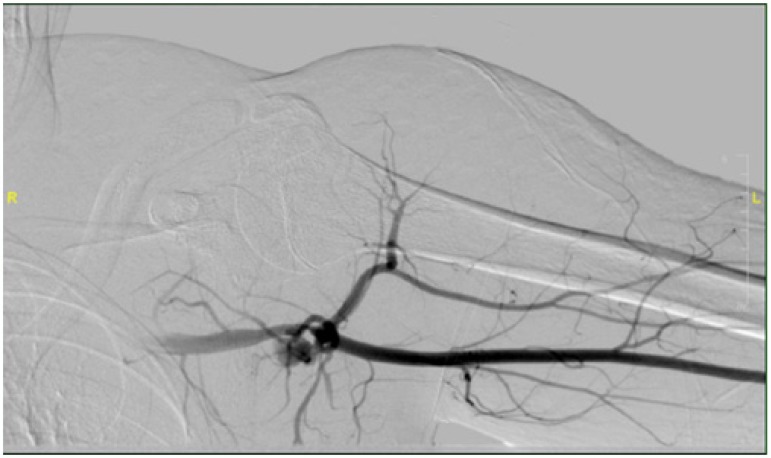
Angiogram showing contrast extravasations from the left axillary artery.

**Fig. 2:**
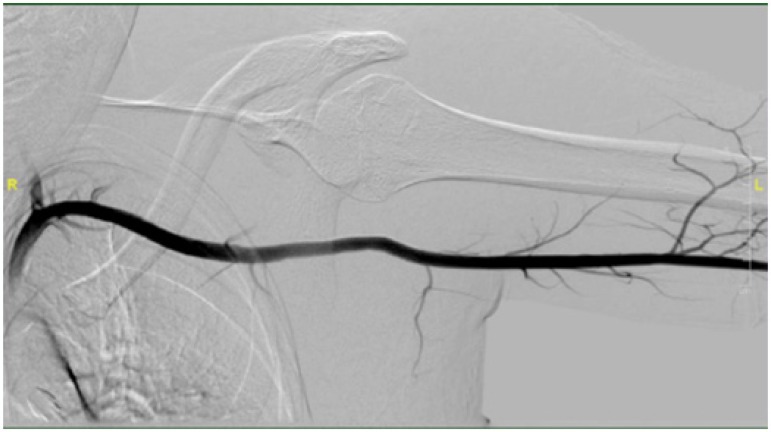
Completion angiography showing the patent-treated axillary artery after the injury.

A computed tomography angiogram of the chest revealed a distinct 5 x 5-mm pseudo-aneurysm in the third part of the axillary artery just proximal to the posterior origin of the circumflex humeral artery. A well-defined regional hematoma was adjacent to that segment of the artery, and no active bleeding was noted. The left brachial artery was accessed in the angio suite, confirming the computed tomography findings with diagnostic angiography. Some contrast extraversion was also observed (Figure 1). Using a 7-F sheath, one 6 x 50 mm VIABAHN^®^-covered stent (W. L. Gore & Associates, Flagstaff, Arizona, USA) was placed over the injury site to cover the origin of the posterior circumflex humeral artery. A 6×100-mm non-compliable balloon was inflated through the stent to ensure complete sealing of the arterial segment. Finally, completion angiography revealed exclusion of the pseudo-aneurism and patent blood flow (Figure 2).

The patient was discharged 3 days after the procedure with take-home medication of 81 mg of aspirin orally once a day. During the 1-month follow-up, duplex scanning revealed normal flow velocities at the patent stent. After 6 months, the patient underwent brachial plexus repair, and a cast was applied postoperatively. During the sixth follow-up visit (8 months post-procedure), he was readmitted because the pulse in his left radial and brachial arteries was not palpable. An urgent duplex scan showed in-stent stenosis with a velocity of 385 cm/s. The next day, a diagnostic angiograph confirmed these findings and also revealed severe stenosis in the stent with a smooth outline and a long segment of arterial stenosis distal to the stent. Balloon angioplasty of the stent and a distal arterial segment were performed using a 6 x16-mm stent as well as a completion angiogram. The patient was then instructed to take 75 mg of clopidogrel daily in addition to aspirin. Clopidogrel was discontinued 3 months after the second procedure. Monthly duplex scanning conducted for four months, then twice yearly, and then yearly revealed normal velocities in the left upper limb arteries.

## DISCUSSION

Endovascular procedures are accepted as management options for penetrating traumatic injuries. Traditionally, open exposure and vessel repair are performed through sternotomy or thoracotomy exposure^[1-2]^. These procedures are time-consuming and pose a risk of collateral injury to the surrounding neurovascular structure. Endovascular procedures have overcome the problems associated with open exposure procedures and are being adopted more often. In the last 10 years, the rate of trauma-associated deaths has increased faster than that of deaths among the general population^[5-7]^, and penetrating trauma accounts for a significant fraction of these deaths. Traumatic artery injuries, such as injuries in the axillosubclavian region, have high rates of morbidity and mortality^5,6)]^, This high rate of morbidity and mortality could be explained because of the need for extensive dissection required in such region on top of the nature of the injury. Under these conditions, endovascular procedures benefit patients much more than traditional exposure and open repair. The Endovascular Skills for Trauma and Resuscitative Surgery Working Group reported that endovascular procedures were successful in 96.9% of patients.^[4]^ Branco et al.^[5]^ analyzed the positive impact of endovascular therapy in patients with sustained axillosubclavian arterial injuries and found a statistically significant difference in mortality: 5.6% for endovascular repair *versus* 27.8% for open repair. These authors also showed that patients treated with endovascular repair tended to have lower rates of complications, particularly surgical-site infections and sepsis.

We used endovascular procedures to treat a penetrating axillary artery injury. More importantly, our long-term cautious therapeutic plan saved the patient's life and afforded him high-quality health. Endovascular procedures have recently been introduced as treatment options for traumatic artery injury and minimized injury to surrounding neurovascular structures and blood loss. These therapeutic methods are characterized by better postoperative morbidity compared with open repair, as clearly shown in this case. We demonstrated that proper adoption of endovascular procedures can lead to a durable repair of a brachial artery injury in young patients. Overall complication rates in these procedures can be high^[4]^; therefore, long-term management care is needed after these procedures. We expect significant improvements for the treatment of vascular injuries as proper management and more endovascular procedures are adopted.

## CONCLUSION

Injury to axillary arteries can be managed via surgery. Endovascular management may yield some better outcomes than open repairs-operating time, local dissection, and decreased potential of injuring other structures. In our case, a proper durable repair of an axillary artery injury was achieved by adopting endovascular procedures, and the stent was successfully used with normal vascularity for as long as 6 years post-procedure.

**Table:** 

Authors' roles & responsibilities	
AA	Substantial contributions to the conception of the work; drafting the work important intellectual content; agreement to be accountable for all aspects of the work; final approval of the version to be published
TN	Agreement to be accountable for all aspects of the work; final approval of the version to be published
AA	Agreement to be accountable for all aspects of the work; final approval of the version to be published
MI	Agreement to be accountable for all aspects of the work; final approval of the version to be published
TAA	Agreement to be accountable for all aspects of the work; final approval of the version to be published
